# Integrated Longitudinal and Lateral Networked Control System Design for Vehicle Platooning

**DOI:** 10.3390/s18093085

**Published:** 2018-09-13

**Authors:** Chedia Latrech, Ahmed Chaibet, Moussa Boukhnifer, Sébastien Glaser

**Affiliations:** 1 Institut VEDECOM, 77 Rue des Chantiers, 78000 Versailles, France; 2 ESTACA, 12 Rue Paul Delouvrier, 78180 Montigny-le-Bretonneux, France; ahmed.chaibet@gmail.com (A.C.); moussa.boukhnifer@gmail.com (M.B.); 3 Centre for Accident Research and Road Safety (CARRS-Q), Queensland University of Technology (QUT), Brisbane city, QLD 4000, Australia; sebastien.glaser@qut.edu.au

**Keywords:** platoon control, vehicle longitudinal control, vehicle lateral control, fuzzy control, linear matrix inequality, time-varying delay

## Abstract

This paper investigates platoon control of vehicles via the wireless communication network. An integrated longitudinal and lateral control approaches for vehicle platooning within a designated lane is proposed. Firstly, the longitudinal control aims to regulate the speed of the follower vehicle on the leading vehicle while maintaining the inter-distance to the desired value which may be chosen proportional to the vehicle speed. Thus, based on Lyapunov candidate function, sufficient stability conditions formulated in BMIs terms are proposed. For the general objective of string stability and robust platoon control to be achieved simultaneously, the obtained controller is complemented by additional conditions established for guaranteeing string stability. Furthermore, constraints such as actuator saturation, and controller constrained information are also considered in control design. Secondly, a multi-model fuzzy controller is developed to handle the vehicle lateral control. Its objective is to maintain the vehicle within the road through steering. The design conditions are strictly expressed in terms of LMIs which can be efficiently solved with available numerical solvers. The effectiveness of the proposed control method is validated under the CarSim software package.

## 1. Introduction

The platooning of autonomous vehicles within a designated lane offers many favors, such as conduct safety and welfare, reducing fuel consumption and air pollution, and improving the throughput within a designated lane [1,2]. These profits are provided by ensuring that all the cars automatically adjust their own speeds as to steady a desired inter-vehicle distance [3,4,5]. Due to this, a lot of research works on platoon control, which introduced many well-known topics in terms of stability, platoon performances, have been elaborated in [6,7].

To ensure platooning control, several communication topologies are developed in the literature [8] and can be classified into three broad categories:

- local control strategy (LCS): based on a local context, the convoy is controlled from near-approach.
- global control strategy (GCS): based on the global context, the convoy is ordered by reference to the leader.
- mixed control strategy (MCS): taking into account the complementarities of the two LCS and GCS methods, a mixed approach can be developed.

The easely control architecture to enable platooning is the local strategy, where platooning is enabled by inter-vehicle communication in addition to on-board sensors. In [9], an adaptive heterogeneous platoon control method was derived using local topology with inter-vehicle time gaps smaller than one second.

Optimizing the interdistances between vehicles is principal to alleviate traffic jam within a designated lane. Constant Distance (CD) policy and the constant time headway (CTH) policy are the two policies for the longitudinal control of platoons. In [2], a new spacing policy called SSP was proposed. However, CTH is the simplest and most common variable spacing policy [3,10]. Compared with the CD policy [11], variable time headway can vary linearly with speed, therefore it can be large to avoid collision [12]. Thus, the best choice is variable spacing because the autonomous vehicle must be able to adjust its speed and to maintain a safe distance behind the front vehicles, in such a way that the autonomous vehicle can stop safely in case of emergency. Nevertheless, the need to grow traffic density urged for novel control methods that insure chain stability for short time gaps.

In many studies [11,13], the vehicle platoons are modelled as one-dimensional systems controlled in the longitudinal direction relies on some assumptions. However, in our contribution, we consider also the lateral dynamic in order to include the lane change maneuver representing the real conditions of road traffic. Therefore, to allow the road convoy within a designated lane, control of both the longitudinal and lateral motion of the vehicle is required. The primary goal of the lateral control is to ensure convenient tracking performance in spite of the coupling effects due to longitudinal speed variation. In [14], the following cars equipped with low-level longitudinal (controlling speed) and lateral (controlling steering) control systems, travel in a platoon with predefined gaps between them. In order to linearize the lateral dynamic, TS fuzzy modeling is adopted [15,16,17], and the proposed automatic lane keeping method can handle a large change area of vehicle speed. Furthermore, Taylor’s approximation process is used to significantly minimize the computational perplexity of the vehicle TS fuzzy model [18].

Nowadays, data networking technologies are being extensively applied in automobile applications, which contribute to the advancement of research and development of NCS. The communication components of the control system, such as sensors, controllers and actuators through a network, can strongly reduce system complexity, with thrifty funding. In addition, this network enables efficient sharing of data between vehicles. However, NCS constitute a new class of systems, introducing specific problems related to the presence of delays, the loss of information, or the management of the data flow. These constraints acquire a great importance because they can cause platoon instability. To raise defiance, many results have been developed in consideration of network-induced delay [19,20,21], with emphasis on stability analysis and controller design with constant or variable delays. In [13], vehicles in platoon share data, via VANETs, affected by actuator delay. In [22], they endorsed a low order of Padé approximation for the delays to develop the controller design. Unlike present paper, variable delay is chosen. In this context, only some of the platoon information can be measured directly by local sensors while the rest of the information needs to be transmitted through the network. To solve this problem, a novel control theory has been applied to the networked systems based on ’miscellaneous information feedback’. The contribution of this paper is to consider this aspect of ’miscellaneous information feedback’ which is not dealt with much in literature [21]. It is noteworthy that, in most of the available works, the saturation effects of actuator are not taken into account in control design. This can lead to serious degradation of control performance and, in many cases, the stability may be lost [23,24]. In [25], bidirectional platoon control considering actuator saturation and time-varying delay is proposed.

The aim of this paper is to set up an autonomous platoon control framework that takes full consideration of time-varying communication delay and actuator saturation. In the first, we model the platoon system pursuant to longitudinal behavior. In the second part, based on the fuzzy Lyapunov function idea, the design problem is formulated as a set of LMI constraints that guarantees the global stability of the platoon. Then, we propose a robust $H_{\infty }$ control law, for lateral dynamics, based on TS fuzzy approach. The main contributions of the current work, compared to existing ones [11,21,26], can be summarized as follows:

- We investigate the impact of the car group on the circulation flow by using a local architecture for platoon. Compared to the mixed structure adopted in [11], this one just employs data from neighboring vehicles and the car is entirely autonomous—hence it doesn’t need sophisticated sensors.
- We also propose a variable spacing unlike the constant policy utilized in [11,21]. Then, we develop the corresponding dynamic control law, study the individual and the string stability of the platoon and demonstrate the effectiveness and safety of the new approach.
- The robustness of the proposed control law is considered regarding the communication delays. In addition, the comfort of the passenger by saturating actuators (e.g.,the maximum deceleration and the maximum jerk (The acceleration’s time derivative is the best way to exhibit a human comfort criteria.)) is taken into account. This strategy is compared to [26] in order to show its advantages with respect to communication delay in design.
- The integrated longitudinal and lateral autonomous is considered in order to cover lane change maneuver. Using TS fuzzy modeling to represent the lateral vehicle dynamic as in [18], the proposed $H_{\infty }$ lateral controller can handle a large variation range of vehicle speed. This approach reduces the design conservatism.

The paper is organized as follows. Section 2 is dedicated to the longitudinal control design of autonomous vehicle while the lateral control one is proposed in Section 3. Section 4 is devoted to simulation results and shows the impact of wireless communication in individual and string stability using the CarSim software package. Finally, some conclusions have been made and future works will be highlighted.

**Notations**: The notation used throughout is as follows. $A^{T}$ denote the transpose of a matrix *A*. Symbol (•) indicates symmetric entries. $I_{n}$ denotes an n×n dimensional identity matrix and He(A) denotes $A+A^{T}$. $l_{2}$ is the space of square integrable functions over [0,∞), and ${\left||.|\right|}_{2}$ denotes the $l_{2}$-norm.

## 2. Longitudinal Controller Design

### 2.1. Longitudinal Vehicle Dynamics Modelling and Feedback Linearisation

**Figure 1:**
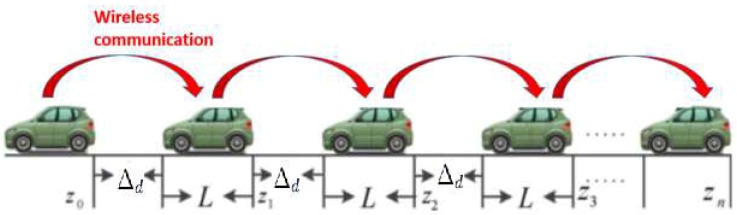
Topological structure of vehicle platooning.

We consider a platoon composed by *N* vehicles rolling along a single lane. Figure 1 shows the platoon where vehicles are fixed as a sequence, where each vehicle can communicate with its preceding vehicle via wireless communication.

The reference trajectory is imposed by the leading vehicle (the first vehicle of platoon labeled as vehicle 0). Our target is to synchronize the dynamics of all vehicles of the platoon to the reference behavior imposed by leader.

In this paper, we focus in the local strategy (LCS) with variable spacing which requires data communication between the vehicles. Let $z_{i}$, $v_{i}$ and $a_{i}$ denote the *i*th (*i* = 1,…,*n* − 1) following vehicle’s position, velocity and acceleration (*i* = 0 stands for the lead vehicle). Define the spacing error of the *i*th following vehicle as:

(1) $$\Delta_{i}=z_{i-1}-z_{i}-\Delta_{d}-L.$$

A constant time headway (CTH) spacing policy will be adopted to regulate the spacing between the vehicles. The CTH is implemented by defining the desired safe distance as:

(2) $$\Delta_{d}=h_{d}v_{i}+d_{stop},$$

where $\Delta_{d}$ is the desired vehicle spacing, *L* is the length of vehicle, and $d_{stop}$ is the standstill distance (m). The dynamics of the *i*th following vehicle can be modelled by the following linear differential equations (see e.g., [11] for details):

(3) $$\left\{\begin{array}{cc} \dot{\Delta_{i}} & =v_{i-1}-v_{i}-h_{d}a_{i},\\ {\dot{v}}_{i} & =a_{i},\\ {\dot{a}}_{i} & =-\frac{1}{\varsigma_{i}}a_{i}+\frac{1}{\varsigma_{i}}u_{i}, \end{array}\right.$$

where $\varsigma_{i}$ stands for the “lumped“ time delay of the actuators such as engine time constant.

Define $x\left(t\right)=Col{\left[x_{i}\left(t\right)\right]}_{i=1}^{n-1}$, $u\left(t\right)=Col{\left[u_{i}\left(t\right)\right]}_{i=1}^{n-1}$, $y\left(t\right)=Col{\left[y_{i}\left(t\right)\right]}_{i=1}^{n-1}$, $z_{2}\left(t\right)=Col{\left[z_{2i}\left(t\right)\left(t\right)\right]}_{i=1}^{n-1}$ are, respectively, the state, the control input, the measured, and the constrained output vectors where $x_{i}\left(t\right)={\left[\begin{array}{ccc} \Delta_{i} & v_{i} & a_{i} \end{array}\right]}^{T}$ and $y_{i}\left(t\right)={\left[\begin{array}{ccc} \Delta_{i} & v_{i-1}-v_{i} & a_{i-1}-a_{i} \end{array}\right]}^{T}$. Using system (3), the state space equation of the platoon can be written as

(4) $$\dot{x}\left(t\right)=Ax\left(t\right)+Bu\left(t\right),$$

where

(5) $$\begin{array}{ccc} A=\left[\begin{array}{cccc} A_{h} & 0 & \cdots & 0\\ A_{s} & A_{h} & \cdots & 0\\ \cdots & \ddots & \ddots & \cdots \\ 0 & \cdots & A_{s} & A_{h} \end{array}\right], & B=\left[\begin{array}{cccc} B_{h} & 0 & \cdots & 0\\ 0 & B_{h} & \cdots & 0\\ \cdots & \ddots & \ddots & \cdots \\ 0 & \cdots & 0 & B_{h} \end{array}\right], & \\ A_{h}=\left[\begin{array}{ccc} 0 & -1 & -h_{d}\\ 0 & 0 & 1\\ 0 & 0 & -1/\varsigma_{i} \end{array}\right], & A_{s}=\left[\begin{array}{ccc} 0 & 1 & 0\\ 0 & 0 & 0\\ 0 & 0 & 0 \end{array}\right], & B_{h}=\left[\begin{array}{c} 0\\ 0\\ 1/\varsigma_{i} \end{array}\right]. \end{array}$$

Likewise, the output equations are written as

(6) $$\left\{\begin{array}{cc} y(t) & =C_{y}x\left(t\right),\\ z_{2}\left(t\right) & =D_{z}u\left(t\right), \end{array}\right.$$

where

(7) $$\begin{array}{ccc} C_{y}=\left[\begin{array}{cccc} C_{1} & 0 & \cdots & 0\\ C_{2} & C_{1} & \cdots & 0\\ \cdots & \ddots & \ddots & \cdots \\ 0 & \cdots & C_{2} & C_{1} \end{array}\right], & D_{z}=\left[\begin{array}{cccc} D_{1} & 0 & \cdots & 0\\ 0 & D_{1} & \cdots & 0\\ \cdots & \ddots & \ddots & \cdots \\ 0 & \cdots & 0 & D_{1} \end{array}\right], & C_{1}=\left[\begin{array}{ccc} 1 & 0 & 0\\ 0 & -1 & 0\\ 0 & 0 & -1 \end{array}\right],\\ C_{2}=\left[\begin{array}{ccc} 0 & 0 & 0\\ 0 & 1 & 0\\ 0 & 0 & 1 \end{array}\right], & D_{1}=1/u_{imax}. & \end{array}$$

For each following vehicle, the controller to be designed can be described as follows:

(8) $$u_{i}\left(t\right)=K_{i}y_{i}\left(t\right),$$

where $K_{i}=\left[\begin{array}{ccc} K_{p} & K_{v} & K_{a} \end{array}\right]$ is the controller gain to be determined.

### 2.2. Problem Formulation

#### 2.2.1. Miscellaneous Information Feedback

**Figure 2:**
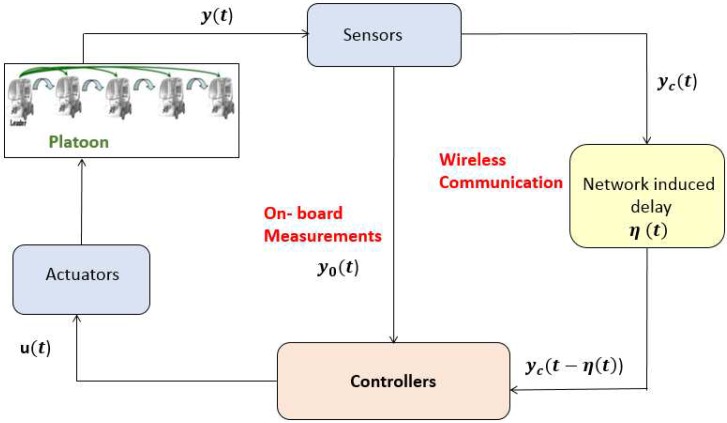
Wireless networked control platoon.

The following Figure 2 represents the structure of networked control platoon. The local control law (8) is achieved by dividing the output vector $y_{i}\left(t\right)={\left[\begin{array}{ccc} \Delta_{i} & v_{i-1}-v_{i} & a_{i-1}-a_{i} \end{array}\right]}^{T}$ into two parts $y_{ci}={\left[\begin{array}{ccc} 0 & v_{i-1}-v_{i} & a_{i-1}-a_{i} \end{array}\right]}^{T}$, and $y_{oi}={\left[\begin{array}{ccc} \Delta_{i} & 0 & 0 \end{array}\right]}^{T}$. Clearly, the spacing error can be directly measured by on-board sensors, whereas the rest of signals are affected by the communication network.

Denote a velocity error $v_{i-1}-v_{i}$ and an acceleration error $a_{i-1}-a_{i}$, the feedback controller gain $K=diag{\left\{K_{i}\right\}}_{1}^{n-1}$, with $K_{i}=\left[\begin{array}{ccc} K_{p} & K_{v} & K_{a} \end{array}\right]$, is split into two parts $K_{o}$ and $K_{c}$, with $K_{o}=diag{\left\{K_{oi}\right\}}_{1}^{n-1}$, $K_{c}=diag{\left\{K_{ci}\right\}}_{1}^{n-1}$, $K_{oi}=\left[\begin{array}{ccc} K_{p} & 0 & 0 \end{array}\right]$ and $K_{ci}=\left[\begin{array}{ccc} 0 & K_{v} & K_{a} \end{array}\right]$.

Then, the overall longitudinal output feedback controller becomes

(9) $$u\left(t\right)=K_{o}y_{o}\left(t\right)+K_{c}y_{c}\left(t\right).$$

#### 2.2.2. Impact of Communication Limitations

Note that the output fragment $y_{c}\left(t\right)$ requires being designed by involving the information of the preceding vehicle successfully broadcasted by the wireless network. Then, there exists a time delay η(t), bounded by $\eta_{1}<\eta \left(t\right)<\eta_{2}$. Thus, for the platoon, the controller given by Equation (9) can be expressed by

(10) $$u\left(t\right)=K_{o}y_{o}\left(t\right)+K_{c}y_{c}\left(t-\eta \left(t\right)\right).$$

**Remark** **1.**

- *Both $\varsigma_{i}$ and η(t) are fixed uniform for both acceleration and brake situations because all of the vehicles are taken to be homogeneous in this work.*
- *Note that more details about the transition from the Equation (9) to the Equation (10) are postponed in [27].*

Substituting (10) into (4), we finally obtain the following platoon system:

(11) $$\left\{\begin{array}{cc} \dot{x}\left(t\right) & =Ax\left(t\right)+BK_{o}y_{o}\left(t\right)+BK_{c}y_{c}\left(t-\eta \left(t\right)\right),\\ y(t) & =C_{o}x\left(t\right)+C_{c}x\left(t\right), \end{array}\right.$$

where

(12) $$\begin{array}{cc} C_{o}=\left[\begin{array}{cccc} C_{3} & 0 & \cdots & 0\\ 0 & C_{3} & \cdots & 0\\ \cdots & \ddots & \ddots & \cdots \\ 0 & \cdots & 0 & C_{3} \end{array}\right], & C_{c}=\left[\begin{array}{cccc} C_{4} & 0 & \cdots & 0\\ C_{2} & C_{4} & \cdots & 0\\ \cdots & \ddots & \ddots & \cdots \\ 0 & \cdots & C_{2} & C_{4} \end{array}\right], \end{array}$$

(13) $$\begin{array}{cc} C_{3}=\left[\begin{array}{ccc} 1 & 0 & 0\\ 0 & 0 & 0\\ 0 & 0 & 0 \end{array}\right], & C_{4}=\left[\begin{array}{ccc} 0 & 0 & 0\\ 0 & -1 & 0\\ 0 & 0 & -1 \end{array}\right]. \end{array}$$

#### 2.2.3. Saturation Effect of Actuator

In real applications, the exact system model is difficult to get and the actuator saturation constantly happens. It should be noted that actuator saturation can deteriorate the platoon’s performance and even cause instability. Thus, in order to guarantee the platoon’s safety and comfort, the following inequality holds:

(14) $$|u_{i}|\leq u_{imax}.$$

Then, after having defined the longitudinal dynamics, we will present the corresponding controller design approach.

#### 2.2.4. The Aim

The control objective of this paper is to achieve the vehicle platoon, with time-varying delay, such that the follower’s velocity can converge to the velocity of the leader asymptotically and each vehicle can maintain a safe inter-vehicle distance to avoid collision with each other. Therefore, we must design a controller (see Equation (9)) for each following vehicle so that the following conditions are satisfied [21]:

1. Individual vehicle stability: the global closed-loop platoon system is asymptotically stable with respect to the communication delay and saturation effects.
2. The vehicle platoon mets the following performance index:
  (15) $$|z_{2i}|\leq 1\;for\;i=1,\cdots ,N.$$
3. String stability: the swings are not magnifying with a vehicle index due to any handling of the head vehicle [10], namely, ∥G(jw)∥<1 for any w>0, where $G\left(s\right)=\Delta_{i}\left(s\right)/\Delta_{i-1}\left(s\right)$; or in the same way, the impulse response g(t) corresponding to G(s) is larger than zero for all *t*.

### 2.3. Single Vehicle Stabilisation

#### Guaranteed Cost Controller Design

Define the time-weighted quadratic cost function as follows:

(16) $$J=\int_{0}^{\infty }\left[x^{T}\left(t\right)Qx\left(t\right)+u^{T}\left(t\right)Ru\left(t\right)\right]dt,$$

where *Q* and *R* are positive definite matrices. The purpose of this subsection is to design a controller which ensure asymptotically stability of system (11) satisfying the performance index $J\leq J^{*}$, where $J^{*}$ is an upper bound of quadratic cost.

We provide the following result for the robust closed-loop system (11).

**Theorem** **1.** *Let scalar $\mu_{1}>0$, and closed-loop networked platoon system (11) is asymptotically stable, if there exist positive matrices P, $T_{1}$, $T_{2}$, $Z_{1}$, $Z_{2}$ and matrices G, $K_{o}$ and $K_{c}$ with appropriate dimensions, such that the following condition holds:*

(17) $$\Xi =\left[\begin{array}{ccccc} \Xi_{11} & \Xi_{12} & Z_{1} & Z_{2} & \Xi_{15}\\ • & \Xi_{22} & 0 & 0 & \Xi_{25}\\ • & • & \Xi_{33} & 0 & 0\\ • & • & • & \Xi_{44} & 0\\ • & • & • & • & \Xi_{55} \end{array}\right]<0,$$

(18) $$\Omega =\left[\begin{array}{ccc} I & {\sqrt{\alpha }}_{1}D_{z}K_{o}C_{o} & {\sqrt{\alpha }}_{2}D_{z}K_{c}C_{c}\\ • & P & 0\\ • & • & P \end{array}\right]>0,$$

where

$$\begin{array}{c} \Xi_{11}=He\left(PA+PBK_{o}C_{o}\right)+T_{1}+T_{2}-Z_{1}-Z_{2}+Q+{\left(KC_{y}\right)}^{T}R\left(KC_{y}\right),\\ \Xi_{12}=\left(PBK_{c}C_{c}\right)+{\left(A+BK_{o}C_{o}\right)}^{T}G,\\ \Xi_{22}=He(G^{T}BK_{c}C_{c}),\\ \Xi_{15}=\mu_{1}{\left(A+BK_{o}C_{o}\right)}^{T}G,\\ \Xi_{25}=-G^{T}+\mu_{1}{\left(BK_{c}C_{c}\right)}^{T}G,\\ \Xi_{33}=-T_{1}-Z_{1},\\ \Xi_{44}=-T_{2}-Z_{2},\\ \Xi_{55}=\eta_{1}^{2}Z_{1}+\eta_{2}^{2}Z_{2}-\mu_{1}He\left(G\right). \end{array}$$

**Proof.**  Define a Lyapunov–Krasovskii function candidate as:

(19) $$\begin{array}{cc} V(t) & =x^{T}\left(t\right)Px\left(t\right)+\int_{t-\eta_{1}}^{t}\;x^{T}\left(s\right)T_{1}x\left(s\right)\;ds+\int_{t-\eta_{2}}^{t}\;x^{T}\left(s\right)T_{2}x\left(s\right)\;ds\\ & +\eta_{1}\int_{-\eta_{1}}^{0}\;\int_{t+s}^{t}\;{\dot{x}}^{T}\left(\upsilon \right)Z_{1}\dot{x}\left(\upsilon \right)\;d\upsilon ds+\eta_{2}\int_{-\eta_{2}}^{0}\;\int_{t+s}^{t}\;{\dot{x}}^{T}\left(\upsilon \right)Z_{2}\dot{x}\left(\upsilon \right)\;d\upsilon ds. \end{array}$$

Employing the Lyapunov function given in (19) and according to zero-value expression obtained from (11), we have:

(20) $$\begin{array}{cc} \dot{V}\left(t\right) & =2x^{T}\left(t\right)P\left(A+BK_{o}C_{o}\right)x\left(t\right)+2x^{T}\left(t\right)PBK_{c}C_{c}x\left(t-\eta \left(t\right)\right)+x^{T}\left(t\right)T_{1}x\left(t\right)\\ & -x^{T}\left(t-\eta_{1}\right)T_{1}x\left(t-\eta_{1}\right)+x^{T}\left(t\right)T_{2}x\left(t\right)-x^{T}\left(t-\eta_{2}\right)T_{2}x\left(t-\eta_{2}\right)\\ & -x^{T}\left(t\right)Z_{1}x\left(t\right)+2x^{T}\left(t\right)\left(t-\eta_{1}\right)Z_{1}x\left(t\right)-x^{T}\left(t-\eta_{1}\right)Z_{1}x\left(t-\eta_{1}\right)-x^{T}\left(t\right)Z_{2}x\left(t\right)\\ & +2x^{T}\left(t-\eta_{2}\right)Z_{2}x\left(t\right)-x^{T}\left(t-\eta_{2}\right)Z_{2}x\left(t-\eta_{2}\right)+\eta_{1}^{2}{\dot{x}}^{T}Z_{1}\dot{x}\left(t\right)+\eta_{2}^{2}{\dot{x}}^{T}Z_{2}\dot{x}\left(t\right)+2[x^{T}\left(t-\eta \left(t\right)\right)G^{T}\\ & +\mu_{1}{\dot{x}}^{T}\left(t\right)G^{T}]\left[-\dot{x}\left(t\right)+Ax\left(t\right)+BK_{o}y_{o}\left(t\right)+BK_{c}y_{c}\left(t-\eta \left(t\right)\right)\right]. \end{array}$$

We can write (20) in the following form:

(21) $$\dot{V}\left(t\right)=\chi^{T}\left(t\right)\left[\begin{array}{ccccc} {\bar{\Xi }}_{11} & \Xi_{12} & Z_{1} & Z_{2} & \Xi_{15}\\ • & \Xi_{22} & 0 & 0 & \Xi_{25}\\ • & • & \Xi_{33} & 0 & 0\\ • & • & • & \Xi_{44} & 0\\ • & • & • & • & \Xi_{55} \end{array}\right]\chi \left(t\right),$$

where ${\bar{\Xi }}_{11}=\Xi_{11}-Q-{\left(KC_{y}\right)}^{T}R\left(KC_{y}\right)$ and

$$\chi \left(t\right)={\left[\begin{array}{ccccc} x^{T}\left(t\right) & x^{T}\left(t-\eta \left(t\right)\right) & x^{T}\left(t-\eta_{1}\left(t\right)\right) & x^{T}\left(t-\eta_{2}\left(t\right)\right) & {\dot{x}}^{T}\left(t\right) \end{array}\right]}^{T}.$$

It is visible from (21) that

(22) $$\dot{V}\left(t\right)\leq -x^{T}\left(t\right)\left(Q+{\left(KC_{y}\right)}^{T}R\left(KC_{y}\right)\right)x\left(t\right)<0.$$

Under the zero-initial condition, integrating (25) over the range [0,∞) yields $\int_{0}^{\infty }\left(Q+{\left(KC_{y}\right)}^{T}R\left(KC_{y}\right)\right)dt<0$.

From the expression of the Lyapunov functional in (19), we obtain that $x^{T}\left(t\right)Px\left(t\right)<\alpha_{1}$ and $x^{T}\left(t-\eta \left(t\right)\right)Px\left(t-\eta \left(t\right)\right)<\alpha_{2}$. As [28], the following inequality holds:

(23) $$\begin{array}{cc} \max_{t>0}{\left|Z_{2}\right|}^{2} & \leq \max_{t>0}{∥x^{T}\left(t\right)C_{o}^{T}K_{o}^{T}D_{z}^{T}D_{z}K_{o}C_{o}x\left(t\right)+x^{T}\left(t-\eta \left(t\right)\right)C_{c}^{T}K_{c}^{T}D_{z}^{T}D_{z}K_{c}C_{c}x\left(t-\eta \left(t\right)\right)∥}_{2}\\ & =\max_{t>0}∥x^{T}\left(t\right)P^{-1/2}P^{1/2}C_{o}^{T}K_{o}^{T}D_{z}^{T}D_{z}K_{o}C_{o}P^{-1/2}P^{1/2}x\left(t\right)\\ & +x^{T}\left(t-\eta \left(t\right)\right)P^{-1/2}P^{1/2}C_{c}^{T}K_{c}^{T}D_{z}^{T}D_{z}K_{c}C_{c}P^{-1/2}P^{1/2}{x\left(t-\eta \left(t\right)\right)∥}_{2}\\ & <\alpha_{1}\sigma_{1max}\left(P^{-1/2}C_{o}^{T}K_{o}^{T}D_{z}^{T}D_{z}K_{o}C_{o}P^{-1/2}\right)+\alpha_{2}\sigma_{2max}\left(P^{-1/2}C_{c}^{T}K_{c}^{T}D_{z}^{T}D_{z}K_{c}C_{c}P^{-1/2}\right), \end{array}$$

where $\sigma_{1max}$ and $\sigma_{2max}$ represent the maximal eigenvalues. The aforementioned inequality leads to the fact that the constraints in (15) are guaranteed, if

(24) $$\alpha_{1}\left(P^{-1/2}C_{o}^{T}K_{o}^{T}D_{z}^{T}D_{z}K_{o}C_{o}P^{-1/2}\right)+\alpha_{2}\left(P^{-1/2}C_{c}^{T}K_{c}^{T}D_{z}^{T}D_{z}K_{c}C_{c}P^{-1/2}\right)-I<0,$$

which is guaranteed by the feasibility of (18). This completes the proof. ☐

**Remark** **2.** *It should be mentioned that the slack variables G added reduce the conservatism of the controller design approach and provide more freedom degrees in the solution space. Furthermore, the objective function (16) offers a trade-off between performance and control effort, according to weighting matrices Q and R.*

**Remark** **3.** *In the following, sufficient conditions (17) are given to ensure asymptotic stability of (11), but they are BMIs and cannot be determined by a convex linear optimization algorithm. Then, we will use a nonlinear optimization based on ’bmibnb’ solver in order to compute gain matrices. Our method is implemented in the Yalmip Toolbox.*

### 2.4. String Stability

A main task of platoon control is to enhance the traffic stream ability while providing safety. Thus, string stability becomes the major performance mainstay, which intends for the spacing errors to reduce as they spread along the vehicle circulation. In an instability case, the conscript ’slinky effect’ will occur and thus cause a potential traffic jam and also a rear-end clash [1,21,25]. It is known that string stability is guaranteed when the transfer function from the spacing error of a vehicle to that of its following vehicle has a magnitude smaller than one second [29]. In this context, an important issue comes from the wide use of wireless communication. The wireless communication channel is an unsettled and very restrictive support, which generally introduces non-negligible time delays. The time delay in wireless communication will largely increase the difficulty to stabilise the platoon, in the presence of the slinky effect. Here, we assume that the communication delay η(t) is uniform and bounded. In [30], optimal adaptive cruise control, with guaranteed string stability considering variable spacing, is addressed. In the actual paper, we have developed techniques to investigate the named string stability property of the vehicle platoon considering both effects of time headway and delay induced by the wireless network. In the above section, considerations have been focused primarily on the stability of each individual vehicles in the platoon system. Here, we tackle the problem of string stability, including the three objectives presented in Section 2.2. In addition, we give results on string stability. The third derivative of Equation (1) gives us

(25) $${\dddot{\Delta }}_{i}\left(t\right)={\dot{a}}_{i-1}\left(t\right)-{\dot{a}}_{i}\left(t\right)-h_{d}{\ddot{a}}_{i}\left(t\right).$$

Substituting (8) into (3), we obtain

$${\dot{a}}_{i}\left(t\right)=-\frac{1}{\varsigma_{i}}a_{i}\left(t\right)+\frac{1}{\varsigma_{i}}K_{i}y_{i}.$$

Combining with (25), the equation of spacing error can be written under the form

(26) $$\begin{array}{cc} \varsigma_{i}{\dddot{\Delta }}_{i}\left(t\right) & =-{\ddot{\Delta }}_{i}\left(t\right)-K_{p}\Delta_{i}\left(t-\eta \left(t\right)\right)-h_{d}K_{p}{\dot{\Delta }}_{i}\left(t-\eta \left(t\right)\right)-K_{v}{\dot{\Delta }}_{i}\left(t-\eta \left(t\right)\right)-K_{a}{\ddot{\Delta }}_{i}\left(t-\eta \left(t\right)\right)\\ & +K_{p}\Delta_{i-1}\left(t-\eta \left(t\right)\right)+K_{v}{\dot{\Delta }}_{i-1}\left(t-\eta \left(t\right)\right)+K_{a}{\ddot{\Delta }}_{i-1}\left(t-\eta \left(t\right)\right). \end{array}$$

Applying the Laplace transform to Equation (26), we can get

(27) $$\begin{array}{cc} G(s) & =\frac{\Delta_{i}\left(s\right)}{\Delta_{i-1}\left(s\right)}=\frac{\left(K_{p}+K_{v}s+K_{a}s^{2}\right)e^{-\eta s}}{\varsigma_{i}s^{3}+s^{2}+\left[K_{p}+\left(K_{v}+h_{d}K_{p}\right)s+K_{a}s^{2}\right]e^{-\eta s}}. \end{array}$$

Based on this transfer function, we have the following result on string stability.

**Theorem** **2.** *For the platoon-spacing error system (26), $|\frac{\Delta_{i}\left(jw\right)}{\Delta_{i-1}\left(jw\right)}|\leq 1$ holds for any w>0, if the following conditions are satisfied:*

(28) $$\left\{\begin{array}{c} \left(a\right)\varsigma_{i}K_{p}-K_{v}-h_{d}K_{p}\leq 0,\\ \left(b\right)K_{a}=\varsigma_{i}\left(k_{v}+h_{d}K_{p}\right),\\ \left(c\right){\left(h_{d}K_{p}\right)}^{2}+2\left(2K_{v}h_{d}K_{p}\right)-2K_{p}\geq 0,\\ \left(d\right)1-K_{a}^{2}+2\eta \left(\varsigma_{i}K_{p}-K_{v}-h_{d}K_{p}\right)\geq 0. \end{array}\right.$$

**Proof.**  First, we write $|\frac{\Delta_{i}\left(jw\right)}{\Delta_{i-1}\left(jw\right)}|$ as

$$G\left(jw\right)=\frac{\Delta_{i}\left(jw\right)}{\Delta_{i-1}\left(jw\right)}=\sqrt{\frac{a}{a+b}},$$

where

(29) $$\begin{array}{cc} a & ={\left(K_{p}-K_{a}w^{2}\right)}^{2}+k_{v}^{2}w^{2},\\ b & =\left[{\left(h_{d}K_{p}\right)}^{2}+2\left(K_{v}h_{d}K_{p}\right)-2K_{p}cos\left(\eta w\right)\right]w^{2}+2\left(\varsigma_{i}K_{p}-K_{v}-h_{d}K_{p}\right)sin\left(\eta w\right)w^{3}\\ & +\left[1+2\left[K_{a}-\varsigma_{i}\left(K_{v}+h_{d}K_{p}\right)\right]cos\left(\eta w\right)\right]w^{4}-2\varsigma_{i}K_{a}sin\left(\eta w\right)w^{5}+\varsigma_{i}^{2}w^{5}. \end{array}$$

Since $a>0,|\frac{\Delta_{i}\left(jw\right)}{\Delta_{i-1}\left(jw\right)}|\leq 1$ hold true, i.e., the platoon is string stable, if b≥0. From (28) and the fact that sin(ηw)≤ηw, we have for w>0 that

(30) $$2\left(\varsigma_{i}K_{p}-K_{v}-h_{d}K_{p}\right)sin\left(\eta w\right)w^{3}\leq 2\eta \left(\varsigma_{i}K_{p}-K_{v}-h_{d}K_{p}\right)w^{4}.$$

Using the condition (28b), we have

(31) $$\begin{array}{cc} b\geq & \left[{\left(h_{d}K_{p}\right)}^{2}+2\left(K_{v}h_{d}K_{p}\right)-2K_{p}cos\left(\eta w\right)\right]w^{2}+\left[1+2\eta \left(\varsigma_{i}K_{p}-K_{v}-h_{d}K_{p}\right)\right]w^{4}. \end{array}$$

Since $\varsigma_{i},K_{p},K_{a}$ and $K_{al}$ are all positive, and the fact that cos(ηw),sin(ηw)≤1, one can obtain

(32) $$\begin{array}{cc} b\geq & \left[{\left(h_{d}K_{p}\right)}^{2}+2\left(K_{v}h_{d}K_{p}\right)-2K_{p}\right]w^{2}+\left[1-K_{a}^{2}+2\eta \left(\varsigma_{i}K_{p}-K_{v}-h_{d}K_{p}\right)\right]w^{4}. \end{array}$$

Thus, if the conditions (28c,d) hold, then b≥0. This completes the proof. ☐

**Remark** **4.** *It should be noted that the conditions for achieving platoon control require combining Theorems 1 and 2. This yields an upper bound for the time delay, that is,* $\eta \leq \frac{1-K_{a}^{2}}{2\left(K_{v}+h_{d}K_{p}\right)-\varsigma_{i}K_{p}}$

## 3. Lateral Controller Design

### 3.1. Bicycle Model

**Figure 3:**
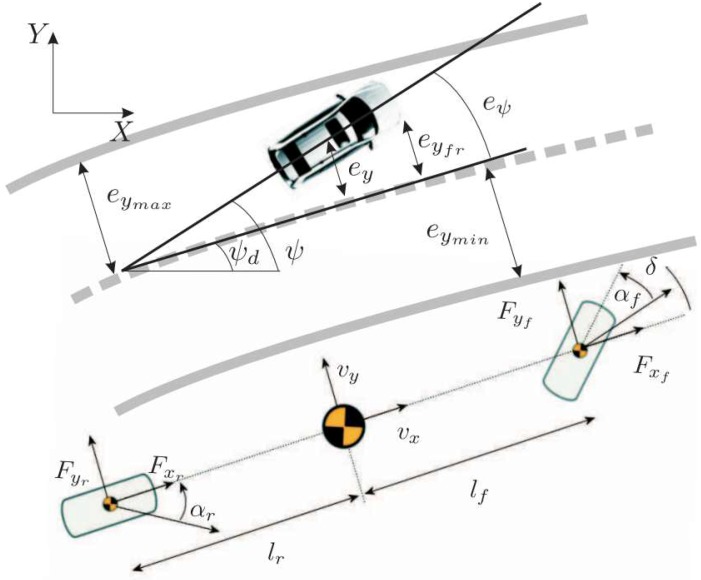
Single track kinematic model of the vehicle.

The simple kinematic vehicle model is used for simpler control of the vehicle dynamics during avoidance screenplay. Then, the single track vehicle model characterizing planar vehicle motion is depicted in Figure 3.

For lateral dynamics, we use the following set of differential equations to describe the vehicle motion within the lane subject to the lateral and yaw dynamics [1]:

(33) $$\left\{\begin{array}{c} \dot{\beta }=\frac{2F_{f}+2F_{r}}{mv}-\dot{\psi },\\ \ddot{\psi }=\frac{2a_{f}F_{f}-2a_{r}F_{r}}{I_{z}},\\ {\dot{e}}_{\psi }=\dot{\psi }-{\dot{\psi }}_{d},\\ {\dot{e}}_{y}=\left(\beta +e_{\psi }\right)v, \end{array}\right.$$

where β denotes the sideslip angle, $F_{f}$ is the cornering force of the two front tires, and $F_{r}$ is the cornering force of the two rear tires. *v* is the longitudinal velocity, $I_{z}$ is the yaw moment of inertia, *m* is the vehicle mass and $\dot{\psi }$ is the yaw rate, where ψ denotes the vehicle orientation. $e_{\psi }$ and $e_{y}$ denote the vehicle orientation and position errors, respectively, w.r.t. the road centerline and $\psi_{d}$ is the orientation of the road centerline. The linear model is the simplest model of the lateral tire forces. It is defined as

(34) $$F_{i}=C_{i0}\alpha_{i},\;i\in f(front),r(rear),$$

where $C_{i0}$ are the cornering stiffness, and $\alpha_{f}$ and $\alpha_{r}$ are the front and rear tire slip angle, respectively, can be approximated as,

(35) $$\left\{\begin{array}{cc} \alpha_{f} & ≅\beta +\frac{a_{f}\dot{\psi }}{v}-\delta ,\\ \alpha_{r} & ≅\beta +\frac{a_{r}\dot{\psi }}{v}, \end{array}\right.$$

where δ denotes the steering angle.

The parameters of the vehicle are given in the following Table 1 [31]:

We compactly rewrite Equations (33)–(35) to form the nonlinear model,

(36) $$\left\{\begin{array}{c} {\dot{\xi }}_{i}\left(t\right)=A_{\xi }\left(v\right)\xi_{i}\left(t\right)+B_{u}\left(v\right)\delta_{i}\left(t\right)+E_{w}W_{i}\left(t\right),\\ y_{1i}\left(t\right)=D_{y}\xi_{i}\left(t\right),\\ Z_{3i}\left(t\right)=D_{z}\xi_{i}\left(t\right), \end{array}\right.$$

where $\xi_{i}\left(t\right)=\left[\begin{array}{cccc} \beta & \dot{\psi } & e_{\psi } & e_{y} \end{array}\right]$, u=δ, $W_{i}\left(t\right)={\dot{\psi }}_{d}$ are the state, input and disturbance vectors, respectively:

$$\begin{array}{ccc} A_{\xi }\left(v\right)=\left[\begin{array}{cccc} -\frac{C_{fi}+C_{ri}}{mv} & -1-\frac{C_{fi}a_{f}-C_{ri}a_{r}}{mv^{2}} & 0 & 0\\ -\frac{C_{fi}a_{f}-C_{ri}a_{r}}{I_{z}} & -\frac{C_{fi}a_{f}^{2}+C_{ri}a_{r}^{2}}{I_{z}v} & 0 & 0\\ 0 & 1 & 0 & 0\\ v & 0 & v & 0 \end{array}\right], & B_{u}\left(v\right)=\left[\begin{array}{c} \frac{C_{fi}}{mv}\\ \frac{a_{f}C_{fi}}{I_{z}}\\ 0\\ 0 \end{array}\right], & E_{w}=\left[\begin{array}{c} 0\\ 0\\ -1\\ 0 \end{array}\right],\\ D_{y}=\left[\begin{array}{cccc} 0 & 1 & 1 & 0 \end{array}\right], & D_{z}=\left[\begin{array}{cccc} 0 & 1 & 1 & 1 \end{array}\right]. \end{array}$$

These nonlinear matrices $A_{\xi }\left(v\right)$, $B_{u}\left(v\right)$ are in function of the vehicle speed *v*,

(37) $$v,\;1/v,\;1/v^{2},\;v_{min}\leq v\leq v_{max},$$

where $v_{min}$ = 2 (m/s) and $v_{max}$ = 30 (m/s). The conventional sector nonlinearity approach will lead to an exact TS fuzzy model with $2^{3}=8$ linear subsystems. However, this accurate approximation would be too expensive in terms of numerical computation for control design. To overcome this problem, Taylor’s approximation method as in [18] is applied to reduce not only the numerical complexity but also the conservatism of the results. Obviously, according to the Taylor’s approximation (first order), we obtain

(38) $$\begin{array}{cccccc} \frac{1}{v} & =\frac{1}{v_{0}}+\frac{1}{v_{1}}\phi_{x}, & v & ≅v_{0}\left(1-\frac{v_{0}}{v_{1}}\phi_{x}\right), & \frac{1}{v^{2}} & ≅\frac{1}{v_{0}^{2}}\left(1+2\frac{v_{0}}{v_{1}}\phi_{x}\right),\\ \phi_{min} & \leq \phi_{x}\leq \phi_{max}, & \phi_{min} & =-1, & \phi_{max} & =1, \end{array}$$

where the measured parameter $\phi_{x}$, called premise variable, is employed to represent the variation of *v* between its lower and upper bounds. Define the two constants $v_{0}$ and $v_{1}$ in (38) as

$$v_{0}=\frac{2v_{min}v_{max}}{v_{min}+v_{max}},\;v_{1}=\frac{2v_{min}v_{max}}{v_{min}-v_{max}}.$$

Using the sector nonlinearity approach after replacing (38) into (36), in order to obtain a TS fuzzy lateral model (33) composed by only two linear subsystems whose matrices are defined as:

(39) $$\begin{array}{c} \Sigma_{v1}:A_{\xi }\left(\phi_{min}\right),\;B_{u}\left(\phi_{min}\right),\\ \Sigma_{v2}:A_{\xi }\left(\phi_{max}\right),\;B_{u}\left(\phi_{max}\right). \end{array}$$

The two corresponding membership functions of this TS fuzzy model are given as follows:

(40) $$h_{1}\left(\phi_{x}\right)=\frac{1-\phi_{x}}{2},\;h_{2}\left(\phi_{x}\right)=1-h_{1}\left(\phi_{x}\right)$$

and satisfying the conditions (41):

(41) $$h_{l}\left(\phi_{x}\right)\geq 0,\;\sum_{l=1}^{r}h_{l}\left(\phi_{x}\right)=1,\;l=1,\ldots 2.$$

Then, the closed-loop system (36) can be written

(42) $$\left\{\begin{array}{c} {\dot{\xi }}_{i}\left(t\right)=\sum_{l=1}^{r}h_{l}\left(\phi_{x}\right)\left[A_{\xi }^{l}\xi_{i}\left(t\right)+B_{u}^{l}\delta_{i}\left(t\right)\right]+E_{w}W_{i}\left(t\right),\\ y_{1i}\left(t\right)=D_{y}\xi_{i}\left(t\right),\\ Z_{3i}\left(t\right)=D_{z}\xi_{i}\left(t\right), \end{array}\right.$$

where the overall lateral output feedback controller is governed by

(43) $$\delta_{i}\left(t\right)=\sum_{l=1}^{r}h_{l}\left(\phi_{x}\right)K_{s}y_{1i}\left(t\right),$$

where $A_{\xi }^{1}$ and $B_{u}^{1}$ correspond to subsystem $\Sigma_{v1}$ and $A_{\xi }^{2}$ and $B_{u}^{2}$ correspond to subsystem $\Sigma_{v2}$.

### 3.2. TS $H_{\infty }$ Design Conditions

In this section, we focus our attention on designing robust $H_{\infty }$ fuzzy SOF controllers gains $K_{i}$ of system (42). To obtain Theorem 3, we were inspired from the results of Theorem 1 in [32], ignoring the effects of interconnection between subsystems and those in [27].

**Theorem** **3.** For given scalars $\eta_{3}>0$, $\eta_{4}>0$, $\mu_{1}$, $\mu_{2}$, and $\mu_{3},$ the closed-loop system (42) is asymptotically stable, if there exist positive matrices $\bar{P}$, ${\bar{Q}}_{1}$, ${\bar{Q}}_{2}$, ${\bar{Q}}_{3}$, ${\bar{Z}}_{1}$, and matrices ${\hat{G}}_{11}>0$, ${\hat{G}}_{21}>0$ and ${\hat{G}}_{22}>0$, and $Y_{s}$, with appropriate dimensions, such that the following conditions hold:

(44) $$\begin{array}{c} {\bar{\Phi }}_{ll}<0, \end{array}$$

(45) $$\begin{array}{c} {\bar{\Phi }}_{ls}+{\bar{\Phi }}_{sl}<0,\;s>l, \end{array}$$

where

(46) $${\bar{\Phi }}_{ls}=\left[\begin{array}{ccccc} {\bar{\Phi }}_{11l} & {\bar{\Phi }}_{12ls} & {\bar{Z}}_{1} & 0 & {\bar{\Phi }}_{15l}\\ • & {\bar{\Phi }}_{22ls} & 0 & 0 & {\bar{\Phi }}_{25ls}\\ • & • & -{\bar{Q}}_{2}-{\bar{Z}}_{1} & 0 & 0\\ • & • & • & -{\bar{Q}}_{3} & 0\\ • & • & • & • & {\bar{\Phi }}_{55} \end{array}\right],$$

$$\begin{array}{cccc} {\bar{\Phi }}_{11l} & ={\bar{Q}}_{1}+{\bar{Q}}_{2}+{\bar{Q}}_{3}+\mu_{1}sym\left(A_{l}\bar{G}\right)-{\bar{Z}}_{1}, & {\bar{\Phi }}_{12ls} & =\mu_{2}{\bar{G}}^{T}{\left(A_{l}\right)}^{T}+\mu_{1}B_{l}Y_{s}D_{y},\\ {\bar{\Phi }}_{22ls} & =\mu_{2}sym\left(B_{l}Y_{s}D_{y}\right)-\left(1-h_{d}\right){\bar{Q}}_{1}, & {\bar{\Phi }}_{15l} & =\bar{P}-\mu_{1}\bar{G}+\mu_{3}{\bar{G}}^{T}{\left(A_{l}\right)}^{T},\\ {\bar{\Phi }}_{25ls} & =-\mu_{2}\bar{G}+\mu_{3}D_{y}^{T}{\left(Y_{s}\right)}^{T}{\left(B_{l}\right)}^{T}, & {\bar{\Phi }}_{55} & =\eta_{1}^{4}{\bar{Z}}_{1}-\mu_{3}sym\left(\bar{G}\right),\\ \bar{G} & =V\left[\begin{array}{cc} {\hat{G}}_{11} & 0\\ {\hat{G}}_{21} & {\hat{G}}_{22} \end{array}\right]V^{T}. \end{array}$$

Then, the desired controller gains are given by $K_{s}=Y_{s}WS{\hat{G}}_{11}^{-1}S^{-1}W^{T}$, where *W*, *S* and *V* are derived from SVD decomposition of $D_{y}$.

### 3.3. Coupling Dynamics

**Figure 4:**
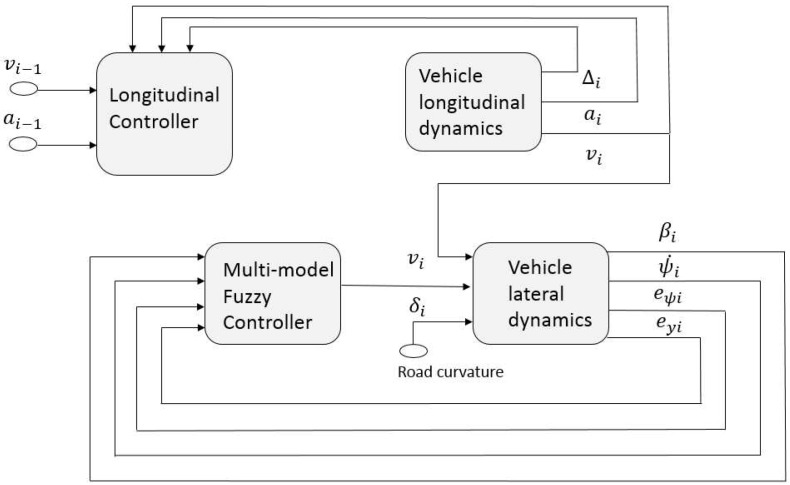
Integrated control system.

In this paper, we combine both the longitudinal and lateral dynamics which are linked by vehicle velocity as shown in Figure 4. Our results clearly differ from existing ones [11,25], which didn’t consider the lateral control system where the platoon will roll just in a straight line. Then, the goal of the lateral control is to maintain the vehicle within the lane through steering.

## 4. Simulation Results

In the following subsections, we evaluate the performances of the proposed control approach. The results are firstly presented for a platoon of six vehicles, which runs in a virtual environment established using the System Build software package in Matlab. After that, simulation results have been carried out using a professional simulator (CarSim) with two vehicles.

### 4.1. Longitudinal Tracking Performance and String Stability

In this subsection, we show how to apply the proposed control method to a vehicle platoon, using Matlab simulation. The desired inter-vehicle distance is variable and depends on velocity. The maximum studied speed is 30 m/s. Three simulation cases will be presented thereafter:

- The following parameters are used in the simulation process: the delay lower bound $\eta_{1}$ = 60 ms, upper bound $\eta_{2}$ = 680 ms, $h_{d}$ = 0.8, $\mu_{1}$ = 0.1, *R* = 1 and $Q=I_{3}$. According to Theorems 1 and 2, we get these controller gains:
  (47) $$K_{p}=0.8471,\;K_{v}=0.9440,\;K_{a}=0.3853.$$
- If we choose $h_{d}=1.5$, $\eta_{1}$ = 60 ms and upper bound $\eta_{2}=800$ ms, we obtain the following controller gains:
  (48) $$K_{p}=0.7627,\;K_{v}=0.2437,\;K_{a}=0.3652.$$
- However, if we neglect transmission delay in design, by Lemma 2 in [26], we can find the following controller gains:
  (49) $$K_{p}=4.9399,\;K_{v}=7.9317,\;K_{a}=3.5481.$$

The components of the initial condition are chosen for the five vehicles as $x_{i}\left(0\right)={\left[\begin{array}{ccc} 0 & 1 & 0 \end{array}\right]}^{T}$, where i=1,⋯,5.

**Figure 5:**
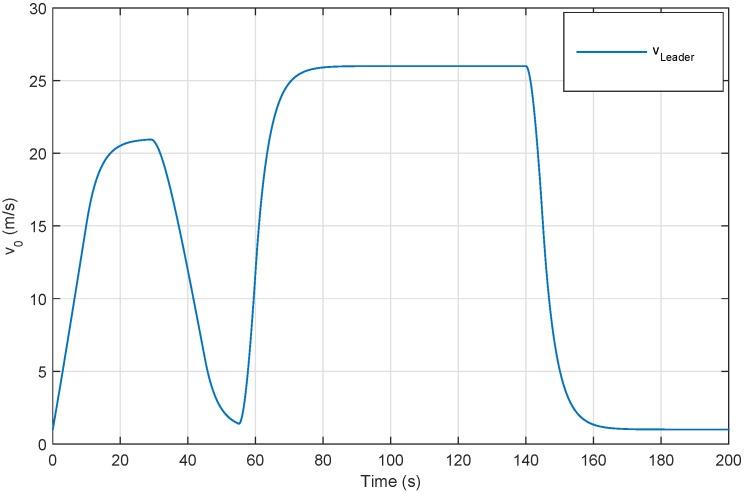
Leader vehicle speed profile.

In order to evaluate the performance of the proposed control approaches, simulations are carried out for a car following scenario by simulating a platoon consisting of six vehicles. The speed profile of leader vehicle is shown in Figure 5 and summarized as follows:

1. Changing the speed of the platoon (from 2 m/s to 20 m/s) at 0 s to verify string stability.
2. Performing an emergency braking at 30 s to satisfy the driver longitudinal ride comfort.
3. Thereafter, the lead vehicle is accelerated and decelerated (hard braking-and-go) to check safety.

All of the following vehicles are controlled to follow the lead vehicle by the proposed controller and controller of Lemma 2 in [26], respectively. At the beginning of the driving scenario, all the vehicles evolve with an initial speed of 2 m/s.

**Figure 6:**
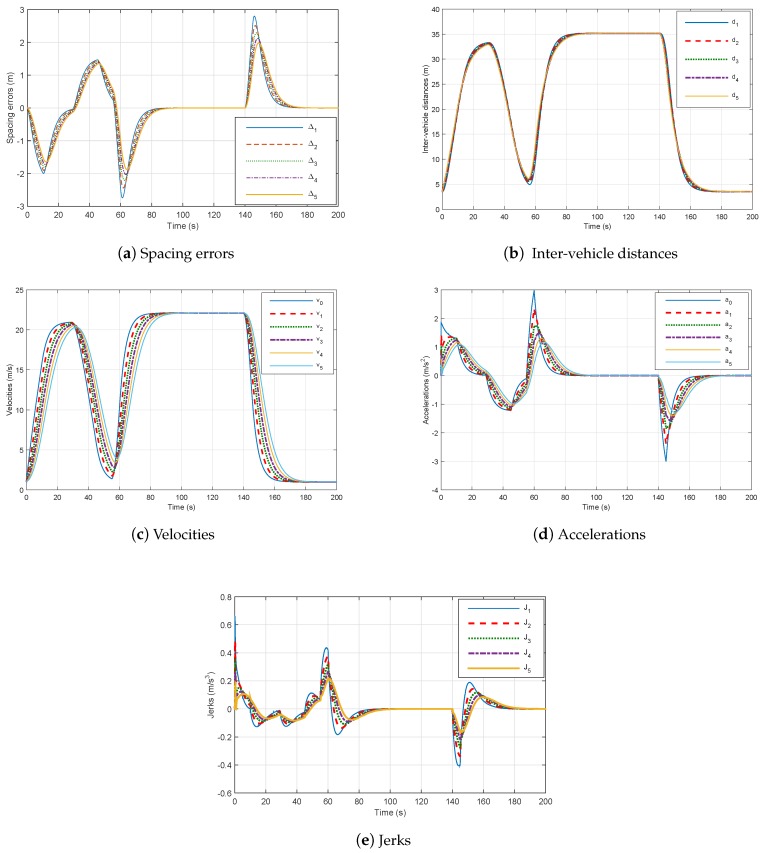
Six-vehicle platoon system under controller (49) without communication network with $h_{d}=0.8$.

Firstly, we denote without considering network communication and using the controllers (49) that the platoon has a stable behavior. The results are clearly illustrated in Figure 6. We can see from Figure 6a that the spacing errors decrease when they propagate through the platoon and the speed of the vehicles converges towards the speed of the leader.

The safety of the platoon in case of an emergency braking is shown in Figure 6c. The inter-vehicle distances are always greater than zero, so no collision occurs. We remark that accelerations for all following vehicles are equal to 3.1 m/s^2^, which corresponds to the comfort acceleration limit. In addition, Figure 6e highlights that the jerk (which is the acceleration’s time derivative is the best way to exhibit a human comfort criteria.) magnitude is reasonably lower than 2 m/s^3^ as defined in [33].

**Figure 7:**
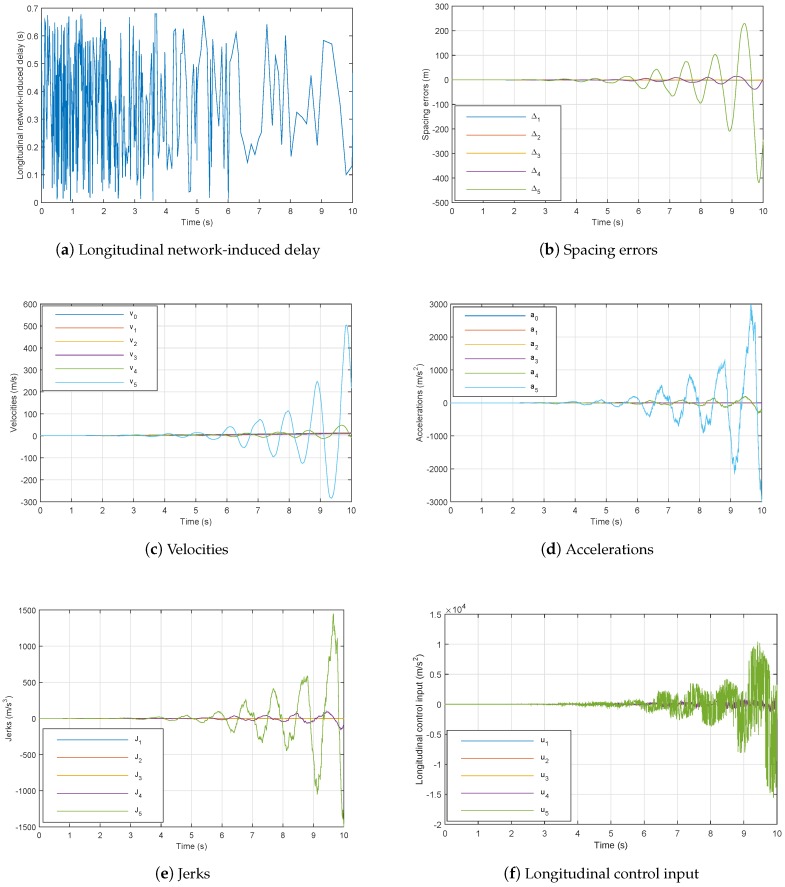
Six-vehicle platoon system under controller (49) through the communication network with $h_{d}=0.8$.

On the contrary, when using the same controller (49) with network communication integration in simulation, the behavior of the platoon becomes unstable as observed in Figure 7. We remark that the inter-vehicle distances are negative and the velocity has bad tracking performance.

**Figure 8:**
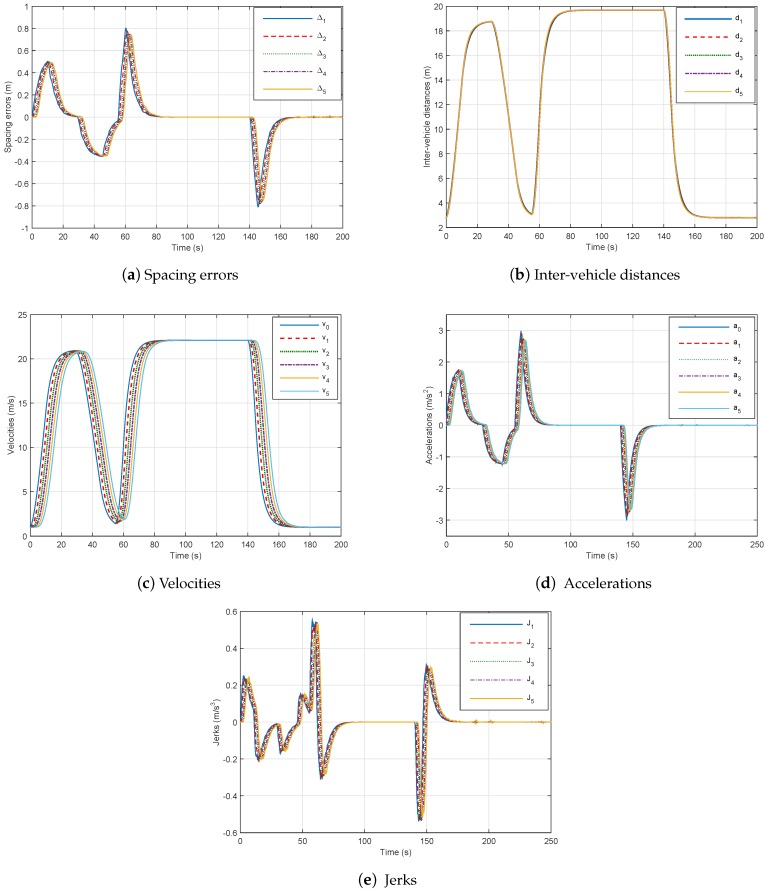
Six-vehicle platoon system under controller (47) through communication network with $h_{d}=0.8$.

**Figure 9:**
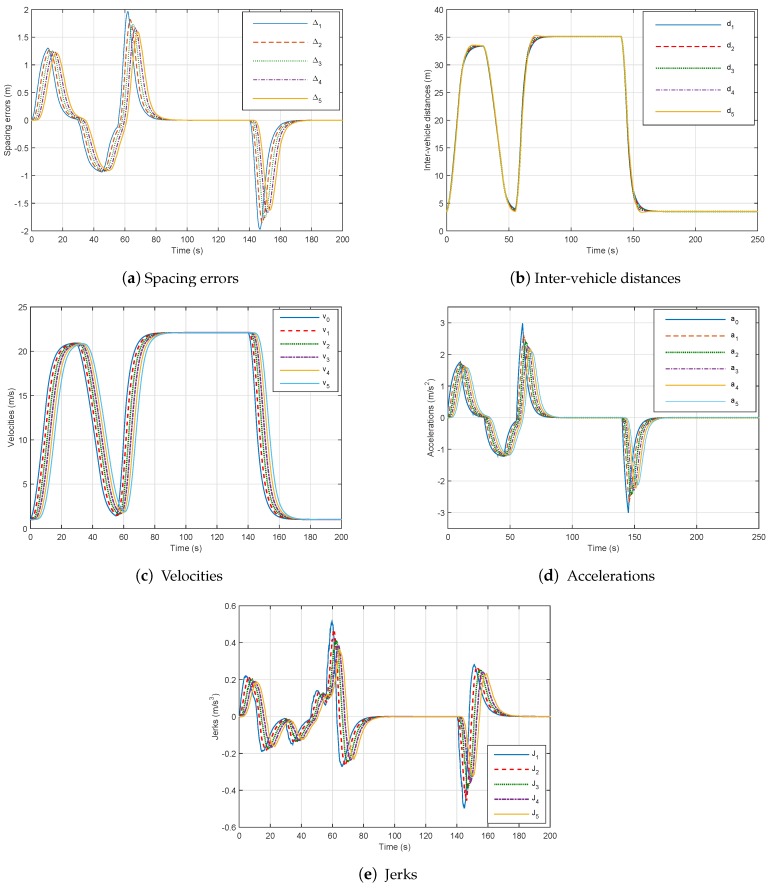
Six-vehicle platoon system under controller (48) through communication network with $h_{d}=1.5$.

Thus, in order to handle the stability and performance of the platoon, despite the effects of the network communication, we have tested the proposed method of Theorem 1 in this paper with considering delay in the control design with (47). The following remarks can be deduced:

- The spacing errors decrease which guarantee string stability as shown in Figure 8a,
- The inter-distances magnitude in the presence of networked communication is positive and smaller than those without delay, which prove the good performance of our controller (48) as in Figure 8b.
- The speed tracking performances are good as well in dynamics as in statics as depicted in Figure 8c.
- The platoon maintains its stability, safety and good performance with controller (47) despite constraints of communication networks.
- Comparing Figure 8 and Figure 9, we can point out that the best performances of platooning, through network communication, can be achieved with a time headway less than 1 s. We remark that with $h_{d}$ = 0.8 s, spacing errors and inter-vehicle distances decrease.

The results indicate that the proposed longitudinal controllers can further improve the platoon system stability and performance, under the effect of communication delays and actuator saturation, with small time headway.

### 4.2. Lateral Control Performance

**Figure 10:**
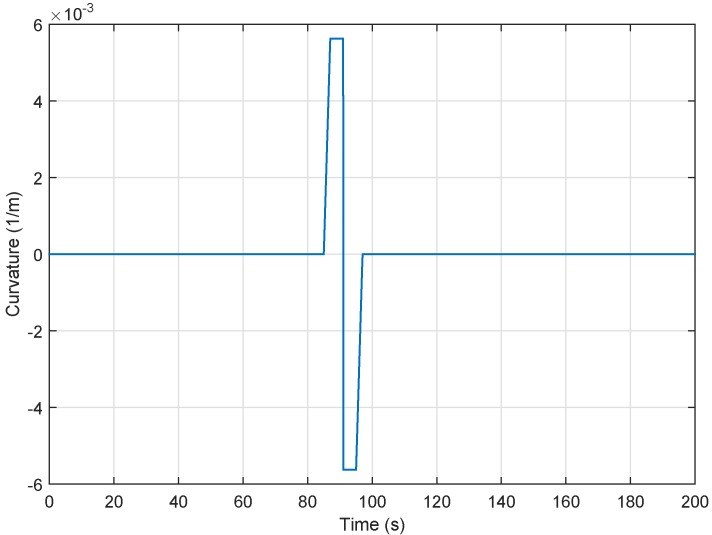
Road curvature.

**Figure 11:**
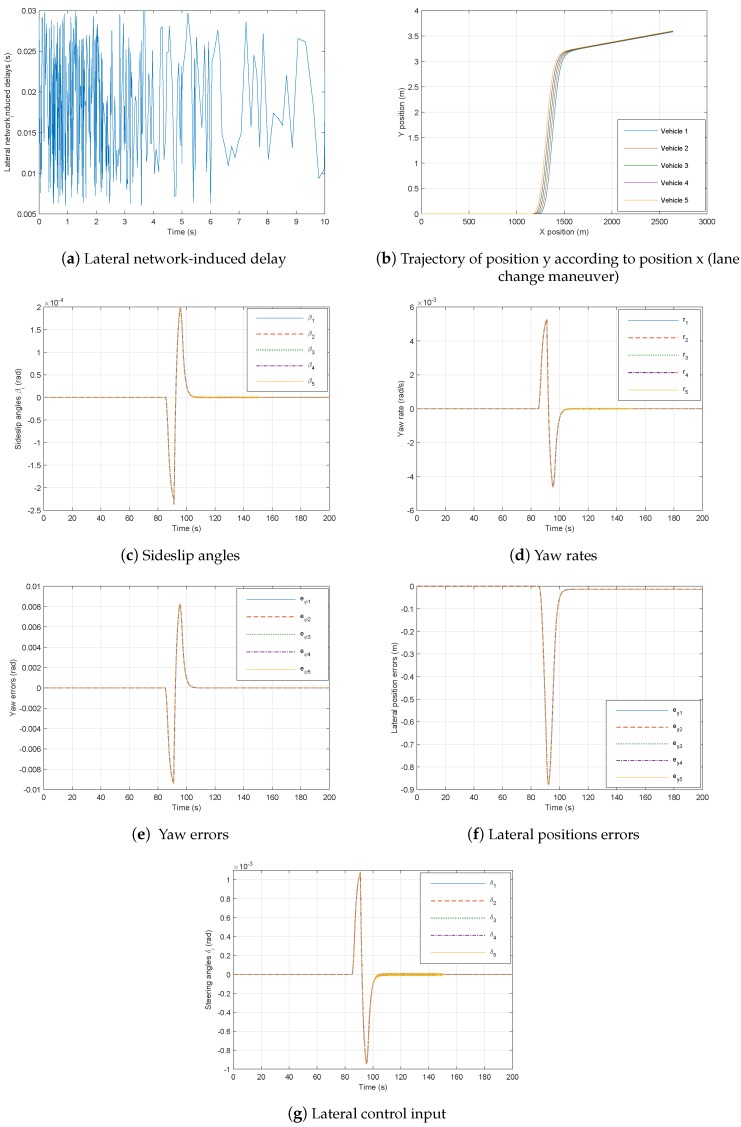
Six-vehicle platoon system under controller (50) through the communication network.

In this subsection, we assess the performance of the proposed lateral control approach. In the lateral control simulation, we consider the curvature of the road traversed by the vehicle platoon shown in Figure 10 and also the trajectories depicted in Figure 11. Then, using the parameters $\mu_{i}=1$ for i=1,…,3, the delay lower bound $\eta_{3}=$ 6 ms, upper bound $\eta_{2}=$ 30 ms, Theorem 3 produces a feasible solution with the following gains matrices:

(50) $$K_{1}=-0.1589,\;K_{2}=-0.1651.$$

For simulation, initial conditions are $\xi_{i}\left(t\right)\left(0\right)={\left[\begin{array}{cccc} 0 & 0 & 0 & 0 \end{array}\right]}^{T}$, where i=1,⋯,5.

Vehicle simulation results have been performed over the lane change maneuver for the fuzzy lateral controller in Figure 11b. Thus, the states and control inputs of the lateral dynamics for followers are illustrated in Figure 11. Obviously, the suggested fuzzy TS controller improves stability and vehicles can track each other with minimal deviation in spite of the communication delays and the rather big variation in longitudinal velocity. As can be seen, the vehicle speed for this scenario strongly varies within its range v∈[2,35]. This clearly justifies the interest of the proposed TS fuzzy model-based control method.

We see from Figure 8 and Figure 11 that the behavior of the platoon becomes stable despite these factors, namely:

1. constraints of communication networks, namely the network-induced delays shown in Figure 7a for longitudinal case and Figure 11a for lateral control which are generated randomly,
2. big variation of the leader longitudinal vehicle speed illustrated in Figure 5.

### 4.3. CarSim Software Validation

CarSim is a professional software dedicated to the simulation of the vehicle dynamics, and it is developed by the ’Mechanical Simulation Corporation’ company. With this simulator, any vehicle’s driving test on a test track or on the road can be simulated before the actual real test. Thus, we can virtually reproduce different driving situations and test the behavior of the vehicle and its reaction to different maneuvers (lane change, slalom, acceleration, slope, etc.). CarSim has five main parts that allow you to choose the simulation parameters, the test conditions and the animation as well as the illustration of the results:

- Vehicle Parameters: This block is used to define several physical parameters of the vehicle (dimensions, engine, tires, bodywork and mathematical models that represent the tire/ground contact forces and suspension forces exerted on the vehicle, etc.).
- Test conditions: In this block, we can create our own test circuit, choose the maneuver, the state of the road and the aerodynamic forces.
- Code Generator: This block is used to generate a block diagram that can be used in various mathematical calculation tools Matlab/Simulink, labVIEW, dSPACE.
- Animation: once the program has been compiled, this block allows for visualizing the maneuver on a 3D video.
- Visualization: For each test, this block allows you to record and plot variables and measures chosen by the user.

CarSim has a standard interface to Matlab/Simulink allowing the co-simulation between them.

**Figure 12:**
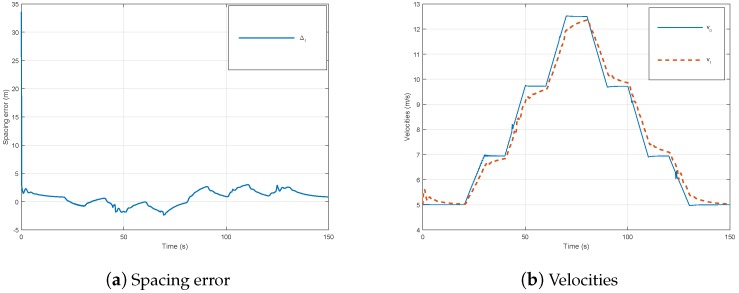

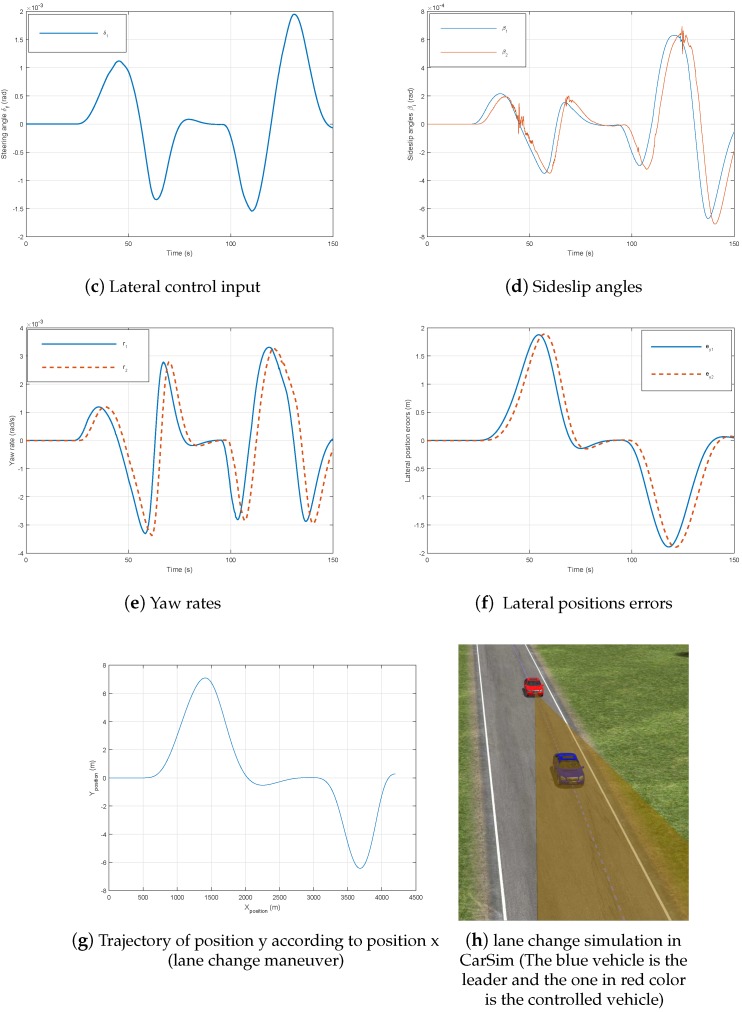
Two-vehicle platoon system under controllers (47) and (50) through the communication network.

Thus, the platooning approach is verified by CarSim in an obstacle avoidance scenario (Figure 12g). A double lane change test (Figure 12h) is conducted with steering wheel angles depicted in Figure 12c. In this simulation, the vehicles are driven at a variable speed (Figure 12b). We can see from the results shown in Figure 12a–g the good performance and efficiency of the proposed approach despite communication delays. In fact, the controlled vehicle managed to follow its preceding vehicle (leader).

## 5. Conclusions

In this paper, the design of integrated controllers for autonomous vehicles has been addressed. Both longitudinal control and lateral control approaches were developed and analyzed. On the one side, vehicle longitudinal control was designed and robustness of the control laws regarding the communication delays and actuator saturation was also dealt. A new platoon model considering a variable inter-vehicle distance proportional to vehicle velocity has been defined. The objective of the proposed controller is to regulate the speed of the follower vehicle while keeping the inter-distance to the desired value. On the other hand, the lateral control was studied aiming to maintain the vehicle within the road through steering. Then, an integrated control structure was proposed, and the longitudinal and the lateral controllers were combined for the fully automated vehicle control. The simulation results showed that the proposed controllers were able to perform accurate longitudinal control and lateral control as well as provide good ride quality.

Our future research topic includes the experimental validation of the proposed approach by urban vehicles.

## Figures and Tables

**Table 1:** Vehicle parameters.

Symbols	Value	Units	Meaning
$I_{z}$	2000	Kg m^2^	Yaw moment of inertia
*m*	1500	Kg	Vehicle mass
$a_{f}$	1.3	m	Distance from COG to front wheel center
$a_{r}$	1.7	m	Distance from COG to rear wheel center
$C_{f0}$	100,000	N/rad	Nominal cornering stiffness of front tire
$C_{r0}$	120,000	N/rad	Nominal cornering stiffness of rear tire
$F_{f}$	-	N	Front tyre cornering force
$F_{r}$	-	N	Rear tyre cornering force
$\alpha_{f}$	-	rad	Front tyre slip angle
$\alpha_{r}$	-	rad	Rear tyre slip angle

## Abbreviations

The following abbreviations are used in this manuscript:

NCS	Networked Control System
TS	Takagi Sugeno
LMI	Linear Matrix Inequality
BMI	Bilinear Matrix Inequality
SOF	Static Output Feedback
SSP	Safety Spacing Policy
VANET	Vehicular Ad-hoc NETworks
COG	Center Of Gravity

## References

[1] Kianfar R., Ali M., Falcone P., Fredriksson J. Combined longitudinal and lateral control design for string stable vehicle platooning within a designated lane. Proceedings of the IEEE 17th International Conference on Intelligent Transportation Systems (ITSC).

[2] Zhao J., Elkamel A. (2009). Integrated Longitudinal and Lateral Control System Design for Autonomous Vehicles. IFAC Proc. Vol..

[3] Kianfar R., Falcone P., Fredriksson J. (2015). A control matching model predictive control approach to string stable vehicle platooning. Control Eng. Pract..

[4] Rahmana M., Abdel-Atyb M. (2017). Longitudinal safety evaluation of connected vehicles platooning on expressways. Accid. Anal. Prev..

[5] Tuchner A., Haddad J. (2017). Vehicle platoon formation using interpolating control: A laboratory experimental analysis. Trans. Res. Part C.

[6] Ghasemi A., Rouhi S. (2015). Stability Analysis of a Predecessor-Following Platoon of Vehicles With Two Time Delays. Sci. J. Traff. Trans. Res..

[7] Xiao L., Gang F. (2010). Effects of information delay on string stability of platoon of automated vehicles under typical information frameworks. J. Cent. South Univ. Technol..

[8] Bom J., Thuilot B., Marmoiton F., Martinet P. (2005). A Global Control Strategy for Urban Vehicles Platooning relying on Nonlinear Decoupling Laws. IEEE/RSJ Int. Conf. Intell. Robots Syst..

[9] Abou Harfouch Y., Yuan S., Baldi S. (2017). An Adaptive Switched Control Approach to Heterogeneous Platooning with Inter-Vehicle Communication Losses. IEEE Trans. Control Netw. Syst..

[10] Oncu S., Ploeg J., Van de Wouw N., Nijmeijer H. (2014). Cooperative Adaptative Cruise Control: Network-Aware Analysis of String Stability. IEEE Trans. Intell. Trans. Syst..

[11] Wei Y., Wang L., Ge G. (2017). Event-triggered platoon control of vehicles with time-varying delay and probabilistic faults. Mech. Syst. Signal Process..

[12] Ioannou P., Chien C. (1993). Autonomous intelligent cruise control. IEEE Trans. Veh. Technol..

[13] Wen S., Guo G., Chen B., Gao X. (2018). Event-triggered cooperative control of vehicle platoons in vehicular ad hoc networks. Inf. Sci..

[14] Kamali M., Dennis L., McAree O., Fisher M., Veres S. (2017). Formal verification of autonomous vehicle platooning. Sci. Comput. Program..

[15] Baek J., Park M. (2012). Fuzzy bilinear state feedback control design based on TS fuzzy bilinear model for DC-DC converters. Electr. Power Energy Syst..

[16] Lendek Z., Nagy Z., Lauber J. (2018). Local stabilization of discrete-time TS descriptor systems. Eng. Appl. Artif. Intell..

[17] Ma Y., Chen M. (2016). Finite time non-fragile dissipative control for uncertain TS fuzzy system with time-varying delay. Neurocomputing.

[18] Nguyen A., Sentouh C., Popieul J. (2017). Fuzzy Steering Control for Autonomous Vehicles under Actuator Saturation: Design and Experiments. J. Frankl. Inst..

[19] Gong J., Zhao Y., Lu Z. (2018). Sampled-data vehicular platoon control with communication delay. J. Syst. Control Eng..

[20] Peters A., Middleton R., Mason O. (2014). Leader tracking in homogeneous vehicle platoons with broadcast delays. Automatica.

[21] Guo G., Yue W. (2011). Hierarchical platoon control with heterogeneous information feedback. IET Control Theory Appl..

[22] Xing H., Ploeg J., Nijmeijer H. (2016). Padé Approximation of Delays in Cooperative ACC Based on String Stability Requirements. IEEE Trans. Intell. Veh..

[23] Behera A., Chalanga A., Bandyopadhyay B. (2018). A new geometric proof of super-twisting control with actuator saturation. Automatica.

[24] Wu F., Lian J. (2018). A parametric multiple Lyapunov equations approach to switched systems with actuator saturation. Nonlinear Anal. Hybrid Syst..

[25] Wei Y., Liyuan W. (2017). Robust Exponential *H*_∞_ Control for Autonomous Platoon against Actuator Saturation ans Time-varying Delay. Int. J. Control Autom. Syst..

[26] Wang G., Chen C., Yu S. (2016). Optimization and static output-feedback control for half-car active suspensions with constrained information. J. Sound Vib..

[27] Latrech C., Kchaou M., Guéguen H. (2018). Networked Non-fragile *H*_∞_ Static Output Feedback Control Design for Vehicle Dynamics Stability: A descriptor approach. Eur. J. Control.

[28] Li H., Liu H., Gao H., Shi P. (2012). Reliable Fuzzy Control for Active Suspension Systems with Actuator Delay and Fault. IEEE Trans. Fuzzy Syst..

[29] Swaroop D., Hedrick J. (1996). String Stability of Interconnected Systems. IEEE Trans. Autom. Control.

[30] Liang C., Peng H. (2010). Optimal Adaptive Cruise Control with Guaranteed String Stability. Int. J. Veh. Mech. Mob..

[31] Mondek M., Hromcik M. Linear analysis of lateral vehicle dynamics. Proceedings of the 21st International Conference on Process Control (PC).

[32] Latrach C., Kchaou M., Rabhi A., Elhajjaji A. (2015). Decentralized networked control system design using (TS) fuzzy approach. Int. J. Autom. Comput. (IJAC).

[33] Martinez J., Canudas-de-Wit C. (2007). A Safe Longitudinal Control for Adaptative Cruise Control and Stop-and-Go Scenarios. IEEE Trans. Control Syst. Technol..

